# ukbtools: An R package to manage and query UK Biobank data

**DOI:** 10.1371/journal.pone.0214311

**Published:** 2019-05-31

**Authors:** Ken B. Hanscombe, Jonathan R. I. Coleman, Matthew Traylor, Cathryn M. Lewis

**Affiliations:** 1 Department of Medical & Molecular Genetics, King's College London, London, United Kingdom; 2 Social, Genetic and Developmental Psychiatry Centre, King's College London, London, United Kingdom; 3 Department of Clinical Neurosciences, University of Cambridge, Cambridge, United Kingdom; University of Texas Health Science Center at San Antonio, UNITED STATES

## Abstract

**Introduction:**

The UK Biobank (UKB) is a resource that includes detailed health-related data on about 500,000 individuals and is available to the research community. However, several obstacles limit immediate analysis of the data: data files vary in format, may be very large, and have numerical codes for column names.

**Results:**

ukbtools removes all the upfront data wrangling required to get a single dataset for statistical analysis. All associated data files are merged into a single dataset with descriptive column names. The package also provides tools to assist in quality control by exploring the primary demographics of subsets of participants; query of disease diagnoses for one or more individuals, and estimating disease frequency relative to a reference variable; and to retrieve genetic metadata.

**Conclusion:**

Having a dataset with meaningful variable names, a set of UKB-specific exploratory data analysis tools, disease query functions, and a set of helper functions to explore and write genetic metadata to file, will rapidly enable UKB users to undertake their research.

## Introduction

The UK Biobank (UKB) is a research study of over 500,000 individuals from across the United Kingdom. Participants aged 40 to 69 were invited to attend one of 22 assessment centres between 2006 and 2010. A wide variety of health-related data has been collected at baseline and follow up, including disease diagnoses, life-style information, family history with blood samples taken for genome-wide genotyping and a biomarker panel. Further data are available in follow up studies on different subsets of UK Biobank participants, including imaging, accelerometer measures and mental health information. The resource is open to applications from health scientists (http://www.ukbiobank.ac.uk/register-apply/). An approved application grants investigators access to the UKB data, however, several obstacles limit immediate analysis of the data.

After downloading and decrypting UKB data with the supplied UKB programs, multiple files need to be integrated into a single dataset for analysis. These data files vary in format, may be very large, and have column names based on the numerical field codes of the UKB data showcase, which requires cross-referencing with an associated html file before they can be interpreted. Here, we describe an R package [1] that manipulates these data files. In a single step, the ukbtools package processes the multiple UKB files to create a workspace with a ready-to-use dataset with meaningful column names. The package also includes tools to visualise primary demographic data for quality control (QC) purposes, query disease diagnoses, and explore genetic metadata for genetic association analyses.

## Implementation

### Constructing a UKB dataset

As a first step, users download their data set, decrypt it, and create a "UKB fileset" (.tab, .r, .html) with the supplied UKB programs. An example is included in the ukbtools package vignette "explore-ukb-data" and full details of the download and decrypt process are provided in the "Using UK Biobank Data" documentation (http://biobank.ctsu.ox.ac.uk/crystal/docs/UsingUKBData.pdf).

The data are then ready to be processed with the core ukbtools function ukb_df. The function ukb_df takes two arguments, the stem of the fileset and optionally the full path (if the fileset is in a different location to where it was created with the UKB programs), and returns a dataframe with meaningful column names. Column names are a contraction to snake_case of the full-length variable descriptions in the .html file with all punctuation and R special characters removed. Variable names include a numeric suffix, *_field_index_array*. The index value captures multiple assessments; the array value captures multiple responses. ukb_df_field generates a field code–to–name lookup table.

install.packages("ukbtools")

library(ukbtools)

# To load the included example data

path_to_example_data <- system.file("extdata", package = "ukbtools")

my_ukb_data <–ukb_df("ukbxxxx", path = path_to_example_data)

In the resulting R dataframe, rows correspond to individuals and columns correspond to UKB variables. A unique identifier for each individual is included in the column "eid". All columns from the .tab file are included and categorical variable values are updated with their categorical level information retrieved from the .r file. For a fileset with a 2.6 GB .tab file (approximately 500,000 rows, 2,000 columns), processing takes approximately 46 seconds (MacBook Pro, 2.8 GHz Processor, 16 GB 2133 MHz LPDDR3 Memory).

## Results

After the initial step of creating a UKB dataset, ukbtools provides additional tools to further query and visualize the data, and to retrieve disease diagnoses and explore genetic metadata.

### Primary demographic data

Typically, researchers will focus on a subset of the data, e.g., those individuals meeting some inclusion criteria, or with data available on a particular variable. Visualizing the demographic data for this subset of the UKB can act as a fast QC tool. ukb_context generates a single figure summary of the distribution of primary demographic data for a subset of individuals relative to a reference set. One use for this tool is to establish representativeness. For example, comparing the distribution of the primary demographics of the subset of individuals who have data for a variable of interest to those without data (NA, or missing) for that particular variable. ukb_context also allows users to flexibly specify the comparison groups as a logical vector where TRUE is the subset of interest, and FALSE is the reference.

ukb_context(

my_ukb_data,

subset.var = my_ukb_data$body_mass_index_bmi_f21001_0_0 > = 25,

bar.position = "stack"

)

**Fig 1:**
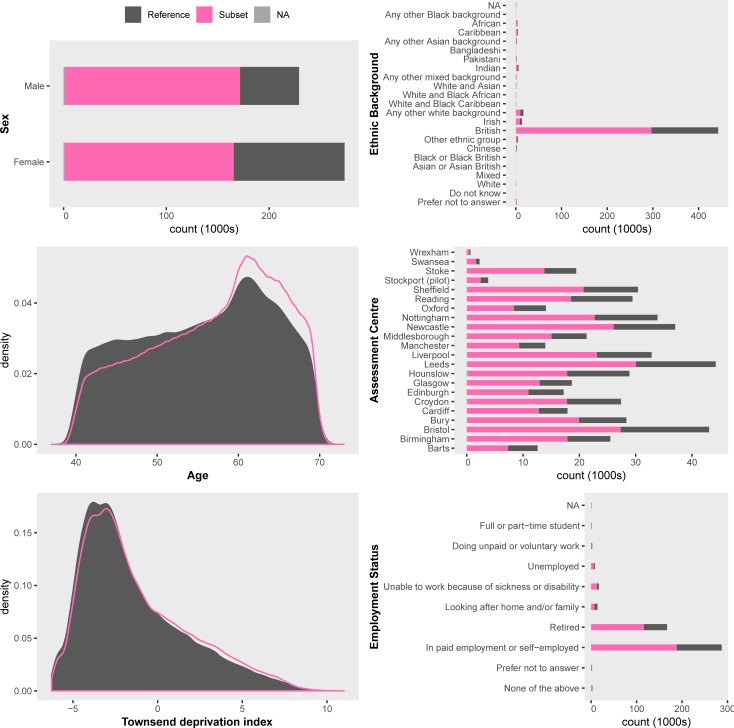
Primary demographic data for a UKB subset of interest. The subset is individuals with BMI > = 25; the reference is BMI < 25. Barplots are displayed as proportions, e.g., Ethnic Background in the top right panel shows about 1/3 of all participants who identified as "Chinese" were overweight compared to about 2/3 of all participants who identified as "British". ukb_context also allows the user to draw barplots as "stacked" or "side-by-side" bars representing counts, which would reveal there were many more "British" participants (442,698) than there were "Chinese" (1,574).

The plot generated by the example above is shown in **Fig 1.** Two alternate plots highlighting insights gained from plotting percentages or counts are included in the supplementary material (**Figures A** and **B** in **S1 File**, and the footnote to **Fig 1**).

### Disease diagnoses

UKB contains rich data from hospital episode inpatient statistics, which can be used for making disease diagnoses. ukbtools includes four diagnosis reference datasets to enable the interpretation of these codes: The International Statistical Classification of Diseases and Related Health Problems (ICD) revision 9 and revision 10 chapters (icd9chapters, icd10chapters) and the ICD-9 and ICD-10 codes (icd9codes, icd10codes). These provide high-level "disease block" information (e.g. chapter 9, disease block I00–I99, Diseases of the circulatory system) and granular diagnosis-specific information (e.g. code I74, Arterial embolism and thrombosis).

Two convenience functions allow the user to query both the codes and the descriptions in the included datasets. Given a particular disease code, ukb_icd_code_meaning retrieves its full description, while ukb_icd_keyword returns all diseases whose descriptions include a particular keyword (actually a regular expression, e.g. "cardio"). The diagnoses of one or more individuals can be retrieved with ukb_icd_diagnosis, which can be used as a QC tool to explain why an individual is an outlier for example, in a particular analysis. A useful exploratory analysis tool is ukb_icd_prevalence, which returns the frequency of an ICD diagnosis code in the UKB dataset. It is possible to explore disease frequency in sub-groups of interest. ukb_icd_freq_by will return the UKB frequency of one or more ICD diagnoses by levels of a reference variable, e.g., sex (male or female). If the variable is continuous, it is divided into *N* (default = 10) approximately equal-sized groups within which ICD diagnosis frequency is calculated.

ukb_icd_freq_by(

my_ukb_data,

reference.var = "body_mass_index_bmi_f21001_0_0"

)

**Fig 2:**
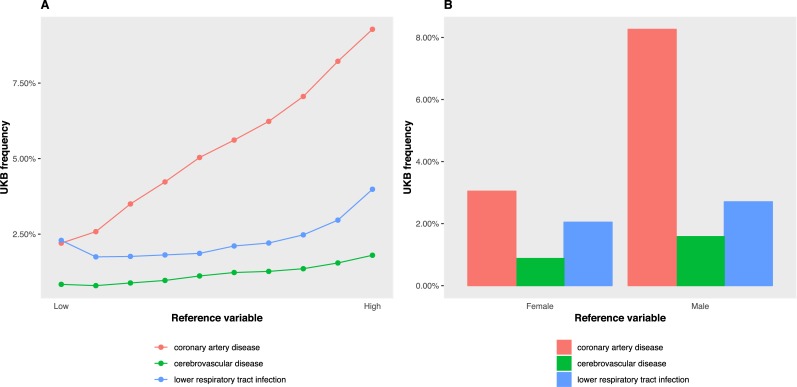
UKB disease frequency by BMI (quantitative) and sex (categorical) reference traits. In panel **A** the reference trait is body mass index, set with the ukb_icd_freq_by argument reference.var = "body_mass_index_bmi_f21001_0_0". Disease frequency is calculated within 10 approximately equal-sized groups (n.group = 10). In Panel **B** the reference trait is sex, reference.var = "sex_f31_0_0". Default ICD codes correspond to the World Health Organization (WHO) 2015 top 3 causes of death globally: "coronary artery disease", "cerebrovascular disease ", and "lower respiratory tract infection". These are supplied to ukb_icd_freq_by as a character vector of regular expressions, icd.code = c("^(I2[0–5])", "^(I6[0–9])", "^(J09|J1[0–9]|J2[0–2]|P23|U04)").

Setting freq.plot = TRUE produces a figure of ICD diagnosis frequency by reference variable. **Fig 2** shows the frequency of disease by to the reference variables BMI (panel **A**) and sex (panel **B**). By default, disease frequencies are plotted for the World Health Organization (WHO) 2015 top 3 causes of death globally: "coronary artery disease", "cerebrovascular disease ", and "lower respiratory tract infection". These can be changed with the option icd.code.

### Genetic metadata

Sample genetic metadata including genotyping array, genetic principal components, inferred sex, heterozygosity (**ukb_sqc_v2.txt**), and relatedness data (**ukbA_rel_sP.txt**) are supplied as separate files. The contents of these files, along with all other genetic data files are described in UKB Resource 531 (https://biobank.ctsu.ox.ac.uk/crystal/refer.cgi?id=531).

ukb_gen_sqc_names provides column names for the sample QC file which comes with no header. ukb_gen_rel_count works with the relatedness file to return either a dataframe of counts of degree of relatedness (duplicates/monozygotic twins, 1^st^-degree, 2^nd^-degree, 3^rd^-degree) or reproduce the relatedness plot (**Figure C** in **S1 File**) on page 15 of the UKB documentation for any subset of the full UKB data (http://www.ukbiobank.ac.uk/wp-content/uploads/2014/04/UKBiobank_genotyping_QC_documentation-web.pdf).

There are many possible ways to remove related individuals from phenotypic data for genetic analyses. Including only those samples "used in the pca calculation" (see **ukb_sqc_v2.txt**) results in an unrelated subset, however, this list is based on the complete dataset and possibly removes more samples than necessary for a specific phenotype of interest. Ideally, users want a maximum independent set, i.e., to remove the minimum number of individuals with data on the phenotype of interest so that no pair exceeds some cutoff for relatedness. ukb_gen_samples_to_remove returns a list of samples to remove in order to achieve a maximal set of unrelateds for a given phenotype (default cutoff > 0.0884, i.e., KING kinship coefficient corresponding to 3^rd^-degree relatedness). For the complete subset of related pairs in which both members have data on the phenotype of interest use ukb_gen_related_with_data.

ukbtools also removes the time-consuming effort of preparing input data for PLINK [2] and BGENIE [3] analyses with a set of read (fam and sample files) and write (phenotype, covariate and exclusions files) functions.

## Conclusions

Having a dataset with meaningful variable names, a set of UKB-specific exploratory data analysis tools, and a set of helper functions to explore genetic QC information and write genetic metadata to file, will rapidly enable UKB users to undertake their research.

## Supporting information

S1 File Supplementary figures. (PDF)

S2 File Example data. A minimal example fileset (r, tab, html) that allows the user to test the read (ukb_df, ukb_df_field) and summarise (ukb_context) functionality. This data is included with the CRAN installation of the package. Access to the data in the package is described in the vignette "Explore UK Biobank data" and package webpage (https://kenhanscombe.github.io/ukbtools/).
(ZIP)

S3 File Bundled package. This bundle contains ukbtools v0.11.3.
(TAR.GZ)

## Abbreviations

UKB: UK Biobank
QC: quality control

## References

[1] R Core Team. 2016 R: A language and environment for statistical computing. R Foundation for Statistical Computing, Vienna, Austria http://www.R-project.org

[2] Chang C C, Chow C C, Tellier L C A M, Vattikuti S, Purcell S M, Lee J J. Second-generation PLINK: rising to the challenge of larger and richer datasets. *GigaScience*. 2015:4 Available from: www.cog-genomics.org/plink/1.9/10.1186/s13742-015-0047-8 PMC4342193 25722852

[3] Bycroft C, Freeman C, Petkova D, Band G, Elliott LT, Sharp K, et al The UK Biobank resource with deep phenotyping and genomic data. Nature. 2018;562: 203–209 10.1038/s41586-018-0579-z 30305743 PMC6786975

