# Supplementary material for: ukbtools: An R package to manage and query UK Biobank data
Source: PLoS One. 2019 May 31;14(5):e0214311. doi: 10.1371/journal.pone.0214311 (PMC6544205; doi:10.1371/journal.pone.0214311)
Supplement: S1 File — (PDF) [file pone.0214311.s001.pdf]

SUPPLEMENTARY FILE S1

ukbtools: An R package to manage and query UK Biobank data

Ken B. Hanscombe[1], Jonathan R.I Coleman[2], Matthew Traylor[3], Cathryn M. Lewis[1,2]

[1]Department of Medical & Molecular Genetics, King's College London, London, United Kingdom, [2]Institute of Psychiatry, Psychology and Neuroscience, Social, Genetic and Developmental Psychiatry Centre, London, United Kingdom, [3]Department of Clinical Neurosciences, University of Cambridge, Cambridge, United Kingdom

Availability: The package is available for installation from the Comprehensive R Archive Network (CRAN), and includes a vignette describing the use of all functionality. For the latest development version, or to log an issue, see https://github.com/kenhanscombe/ukbtools

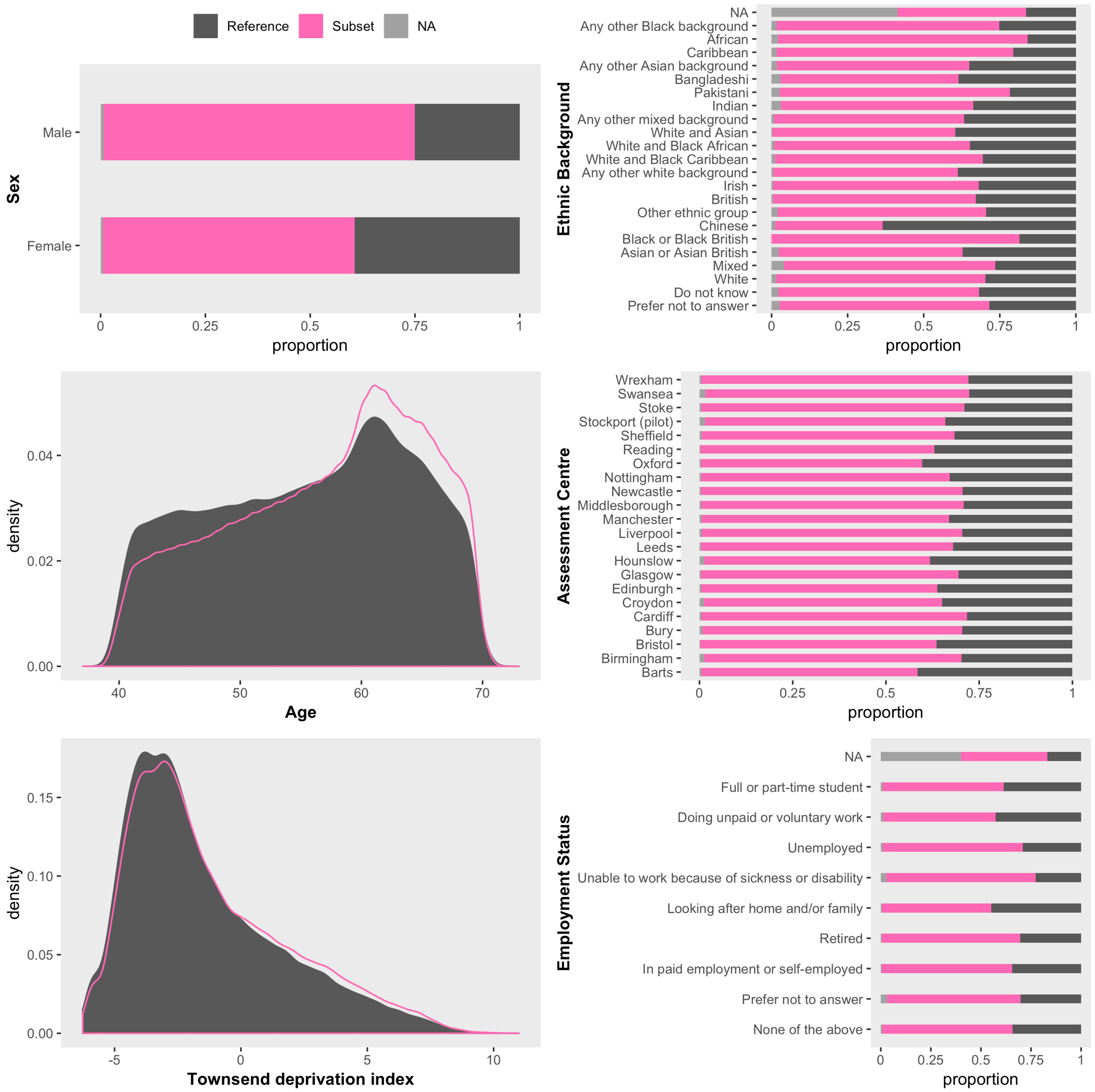

Supplementary Figure A. Primary demographic data for a UKB subset of interest. Alternate demographic plot, `bar.position = "stack"`.

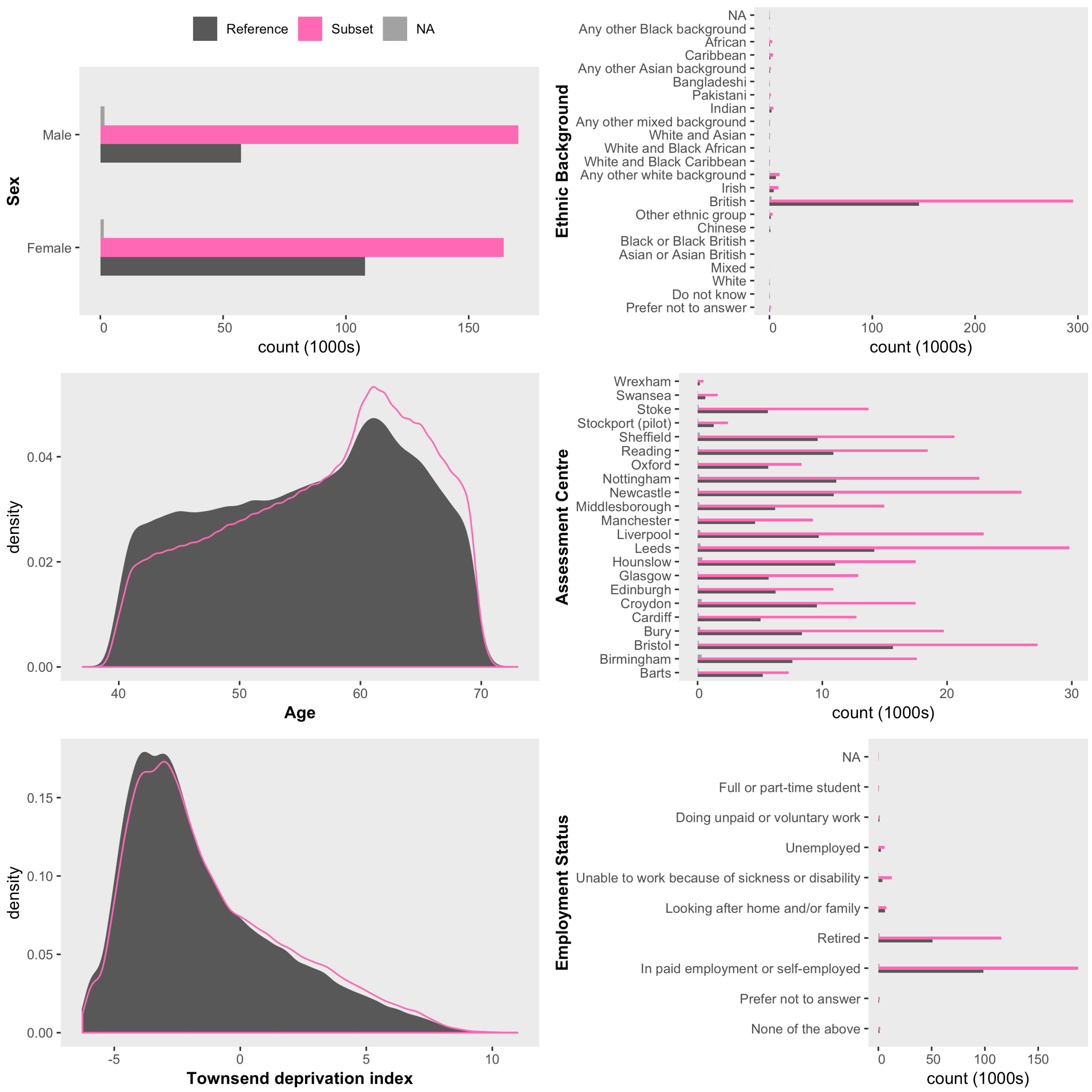

Supplementary Figure B. Primary demographic data for a UKB subset of interest. Alternate demographic plot, `bar.position = "dodge"`

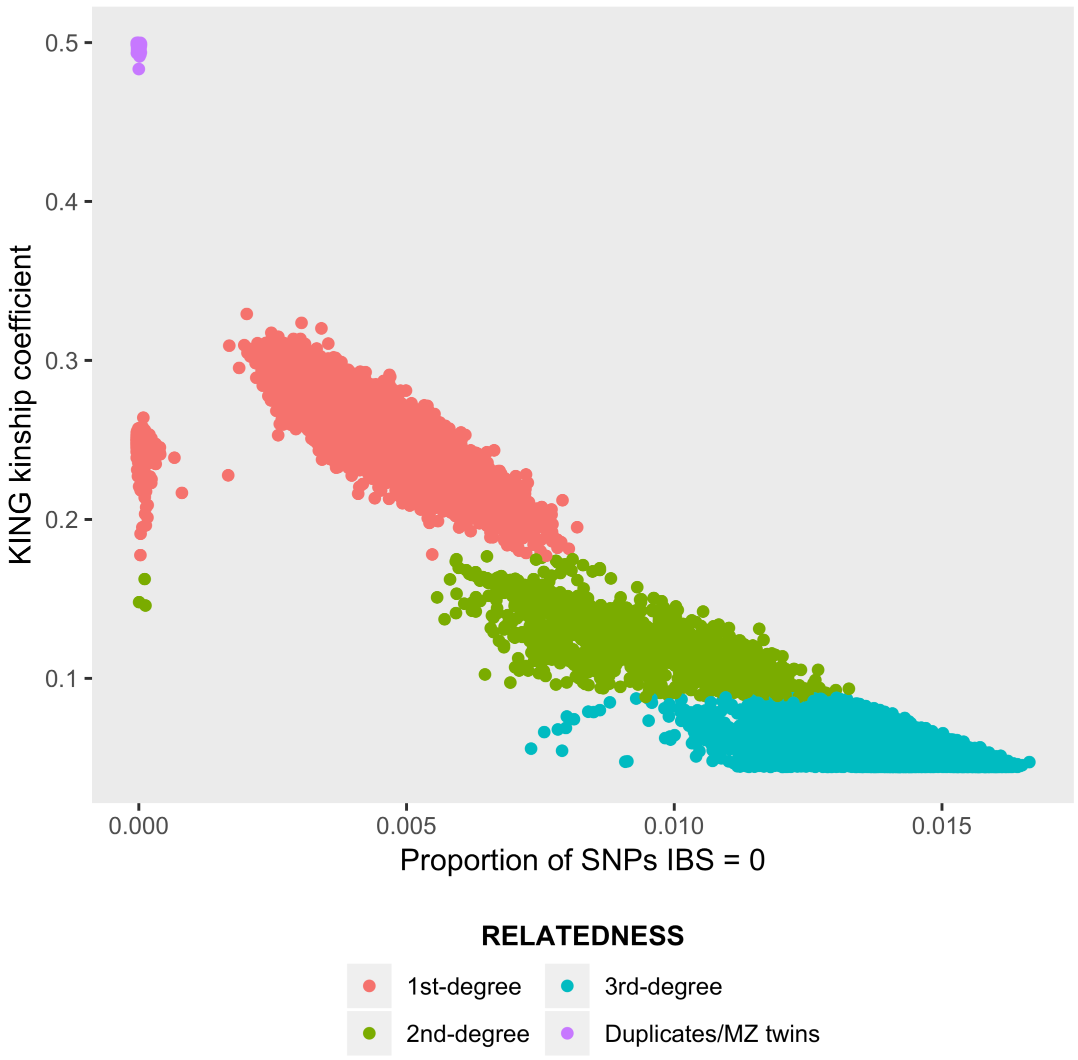

Supplementary Figure C. Genetic relatedness for a UKB subset of interest. Reproduces the relatedness plot on page 15 of the UKB documentation (http://www.ukbiobank.ac.uk/wp-content/uploads/2014/04/UKBiobank_genotyping_QC_documentation-web.pdf) for any subset of the full UKB data. SNP = single nucleotide polymorphism; IBS = identity-by-state
