# Supplementary material for: ukbtools: An R package to manage and query UK Biobank data
Source: PLoS One. 2019 May 31;14(5):e0214311. doi: 10.1371/journal.pone.0214311 (PMC6544205; doi:10.1371/journal.pone.0214311)
Supplement: S2 File — A minimal example fileset (r, tab, html) that allows the user to test the read (ukb_df, ukb_df_field) and summarise (ukb_context) functionality. This data is included with the CRAN installation of the package. Access to the data in the package is described in the vignette "Explore UK Biobank data" and package webpage (https://kenhanscombe.github.io/ukbtools/). (ZIP) [file pone.0214311.s002.zip › ukbxxxx.html]

UK Biobank : Application XXXXY


# UK Biobank : Data Dictionary for Application XXXXY

|  |  |
| --- | --- |
| Date Extracted: | 9999-99-99T99:99:99 |
| Data columns: | 33 |


| Column | UDI | Count | Type | Description |
| --- | --- | --- | --- | --- |
| 0 | eid | 502642 | Sequence | Encoded anonymised participant ID |
| 1 | 31-0.0 | 502642 | Categorical (single) | Sex Uses data-coding 9 comprises 2 Integer-valued members in a simple list. |
| 2 | 54-0.0 | 502642 | Categorical (single) | UK Biobank assessment centre Uses data-coding 10 comprises 24 Integer-valued members in a simple list. |
| 3 | 54-1.0 | 20346 |
| 4 | 54-2.0 | 8000 |
| 5 | 189-0.0 | 502015 | Continuous | Townsend deprivation index at recruitment |
| 6 | 6142-0.0 | 501771 | Categorical (multiple) | Current employment status Uses data-coding 100295 comprises 9 Integer-valued members in a simple list. |
| 7 | 6142-0.1 | 39554 |
| 8 | 6142-0.2 | 6159 |
| 9 | 6142-0.3 | 511 |
| 10 | 6142-0.4 | 35 |
| 11 | 6142-0.5 | 3 |
| 12 | 6142-0.6 | 1 |
| 13 | 6142-1.0 | 20339 |
| 14 | 6142-1.1 | 1774 |
| 15 | 6142-1.2 | 332 |
| 16 | 6142-1.3 | 20 |
| 17 | 6142-1.4 | 1 |
| 18 | 6142-1.5 | 0 |
| 19 | 6142-1.6 | 0 |
| 20 | 6142-2.0 | 8052 |
| 21 | 6142-2.1 | 615 |
| 22 | 6142-2.2 | 119 |
| 23 | 6142-2.3 | 8 |
| 24 | 6142-2.4 | 0 |
| 25 | 6142-2.5 | 0 |
| 26 | 6142-2.6 | 0 |
| 27 | 21000-0.0 | 501743 | Categorical (single) | Ethnic background Uses data-coding 1001 comprises 22 Integer-valued members in a hierarchical tree. |
| 28 | 21000-1.0 | 20339 |
| 29 | 21000-2.0 | 8052 |
| 30 | 21003-0.0 | 502642 | Integer | Age when attended assessment centre |
| 31 | 21003-1.0 | 20002 |
| 32 | 21003-2.0 | 5894 |

### Notes

- UDI - the Unique Data Identifier for an item of data within the UK Biobank repository. The format is field\_id**-**instance\_index**.**array\_index
- Count - the number of non-empty rows present in this dataset.
- Read 999999 rows of data, expected 999999.
- There are -9 distinct data-fields present.
- There are 4 distinct data-codings used.
- UKB reference for dataset: XXXXY/ZZZZ/ZZZZ/9.

### Data-Coding 9

comprises 2 Integer-valued members in a simple list.

|  |  |  |  |  |  |  |  |  |
| --- | --- | --- | --- | --- | --- | --- | --- | --- |
| # | Code | Meaning || 1 | 1 | Male |
| 2 | 0 | Female |

### Data-Coding 10

comprises 24 Integer-valued members in a simple list.

|  |  |  |  |  |  |  |  |  |  |  |  |  |  |  |  |  |  |  |  |  |  |  |  |  |  |  |  |  |  |  |  |  |  |  |  |  |  |  |  |  |  |  |  |  |  |  |  |  |  |  |  |  |  |  |  |  |  |  |  |  |  |  |  |  |  |  |  |  |  |  |  |  |  |  |
| --- | --- | --- | --- | --- | --- | --- | --- | --- | --- | --- | --- | --- | --- | --- | --- | --- | --- | --- | --- | --- | --- | --- | --- | --- | --- | --- | --- | --- | --- | --- | --- | --- | --- | --- | --- | --- | --- | --- | --- | --- | --- | --- | --- | --- | --- | --- | --- | --- | --- | --- | --- | --- | --- | --- | --- | --- | --- | --- | --- | --- | --- | --- | --- | --- | --- | --- | --- | --- | --- | --- | --- | --- | --- | --- |
| # | Code | Meaning || 1 | 11012 | Barts |
| 2 | 11021 | Birmingham |
| 3 | 11011 | Bristol |
| 4 | 11008 | Bury |
| 5 | 11003 | Cardiff |
| 6 | 11024 | Cheadle (revisit) |
| 7 | 11020 | Croydon |
| 8 | 11005 | Edinburgh |
| 9 | 11004 | Glasgow |
| 10 | 11018 | Hounslow |
| 11 | 11010 | Leeds |
| 12 | 11016 | Liverpool |
| 13 | 11001 | Manchester |
| 14 | 11017 | Middlesborough |
| 15 | 11009 | Newcastle |
| 16 | 11013 | Nottingham |
| 17 | 11002 | Oxford |
| 18 | 11007 | Reading |
| 19 | 11014 | Sheffield |
| 20 | 10003 | Stockport (pilot) |
| 21 | 11006 | Stoke |
| 22 | 11022 | Swansea |
| 23 | 11023 | Wrexham |
| 24 | 11025 | Cheadle (imaging) |

### Data-Coding 100295

comprises 9 Integer-valued members in a simple list.

|  |  |  |  |  |  |  |  |  |  |  |  |  |  |  |  |  |  |  |  |  |  |  |  |  |  |  |  |  |  |
| --- | --- | --- | --- | --- | --- | --- | --- | --- | --- | --- | --- | --- | --- | --- | --- | --- | --- | --- | --- | --- | --- | --- | --- | --- | --- | --- | --- | --- | --- |
| # | Code | Meaning || 1 | 1 | In paid employment or self-employed |
| 2 | 2 | Retired |
| 3 | 3 | Looking after home and/or family |
| 4 | 4 | Unable to work because of sickness or disability |
| 5 | 5 | Unemployed |
| 6 | 6 | Doing unpaid or voluntary work |
| 7 | 7 | Full or part-time student |
| 8 | -7 | None of the above |
| 9 | -3 | Prefer not to answer |

### Data-Coding 1001

comprises 22 Integer-valued members in a hierarchical tree.

|  |  |  |  |  |  |  |  |  |  |  |  |  |  |  |  |  |  |  |  |  |  |  |  |  |  |  |  |  |  |  |  |  |  |  |  |  |  |  |  |  |  |  |  |  |  |  |  |  |  |  |  |  |  |  |  |  |  |  |  |  |  |  |  |  |  |  |  |  |
| --- | --- | --- | --- | --- | --- | --- | --- | --- | --- | --- | --- | --- | --- | --- | --- | --- | --- | --- | --- | --- | --- | --- | --- | --- | --- | --- | --- | --- | --- | --- | --- | --- | --- | --- | --- | --- | --- | --- | --- | --- | --- | --- | --- | --- | --- | --- | --- | --- | --- | --- | --- | --- | --- | --- | --- | --- | --- | --- | --- | --- | --- | --- | --- | --- | --- | --- | --- | --- |
| # | Code | Meaning || 1 | 4001 | Caribbean |
| 2 | 3001 | Indian |
| 3 | 1 | White |
| 4 | 2001 | White and Black Caribbean |
| 5 | 1001 | British |
| 6 | 3002 | Pakistani |
| 7 | 2 | Mixed |
| 8 | 4002 | African |
| 9 | 1002 | Irish |
| 10 | 2002 | White and Black African |
| 11 | 3003 | Bangladeshi |
| 12 | 3 | Asian or Asian British |
| 13 | 4003 | Any other Black background |
| 14 | 1003 | Any other white background |
| 15 | 2003 | White and Asian |
| 16 | 3004 | Any other Asian background |
| 17 | 4 | Black or Black British |
| 18 | 2004 | Any other mixed background |
| 19 | 5 | Chinese |
| 20 | 6 | Other ethnic group |
| 21 | -1 | Do not know |
| 22 | -3 | Prefer not to answer |

---

END
