# Supplementary material for: ukbtools: An R package to manage and query UK Biobank data
Source: PLoS One. 2019 May 31;14(5):e0214311. doi: 10.1371/journal.pone.0214311 (PMC6544205; doi:10.1371/journal.pone.0214311)
Supplement: S3 File — This bundle contains ukbtools v0.11.3. (TAR.GZ) [file pone.0214311.s003.tar.gz › ukbtools/inst/doc/explore-ukb-data.html]

Explore UK Biobank data


# Explore UK Biobank data

#### *Ken B. Hanscombe*

#### *2019-05-16*

The **UK Biobank** is a resource that includes detailed health-related and genetic data on about 500,000 individuals and is available to the research community. **ukbtools** removes all the upfront data wrangling required to get a single dataset for statistical analysis, and provides tools to assist in quality control, query of disease diagnoses, and retrieval of genetic metadata.

## Getting started

Download and decrypt your data with the supplied helper programs. To use ukbtools, you need to create a UKB fileset (.tab, .r, and .html):

```
ukb_unpack ukbxxxx.enc key
ukb_conv ukbxxxx.enc_ukb r
ukb_conv ukbxxxx.enc_ukb docs
```

ukb\_unpack decrypts your downloaded ukbxxxx.enc file, outputting a ukbxxxx.enc\_ukb file. ukb\_conv with the r flag converts the decrypted data to a tab-delimited file ukbxxxx.tab and an R script ukbxxxx.r that reads the tab file. The docs flag creates an html file containing a field-code-to-description table (among others).

**Note.** Full details of the data download and decrypt process are given in the Using UK Biobank Data documentation . Updated versions of these helper programs exist. Other than small name changes (underscores removed) they appear to function similarly.

## Installing the package

In R,

```
# Install from CRAN
install.packages("ukbtools")

# Install latest development version
devtools::install_github("kenhanscombe/ukbtools", build_vignettes = TRUE, dependencies = TRUE)
```

## Making a dataset

The function `ukb_df()` takes the stem of your fileset and returns a dataframe with usable column names.

```
library(ukbtools)

my_ukb_data <- ukb_df("ukbxxxx")
```

You can also specify the path to your fileset if it is not in the current directory. For example, if your fileset is in a subdirectory of the working directory called `data`

```
my_ukb_data <- ukb_df("ukbxxxx", path = "/full/path/to/my/ukb/fileset/data")
```

## Making a key

Use `ukb_df_field` to create a field code-to-descriptive name key, as dataframe or named lookup vector.

```
my_ukb_key <- ukb_df_field("ukbxxxx", path = "/full/path/to/my/ukb/fileset/data")
```

**Note.** You can move the three files in your fileset after creating them with `ukb_conv`, but they should be kept together. `ukb_df()` automatically updates the read call in the R source file to point to the correct directory (the current directly by default, or the directory specified by `path`).

> **Memory and efficiency**
>
> To reduce you memory usage, you could save your new UKB dataset with `save(my_ukb_data, file = "my_ukb_data.rda")`. Load the dataset with `load("my_ukb_data.rda")`. A UKB dataset from my largest UKB fileset which included a 2.6 GB .tab file took a little under 2 minutes to create with `ukb_df`. The associated .rda file was 138 MB and loaded in a little under 1.5 mins.

## Multiple downloads

If you have multiple UKB downloads, first read each one in, then merge them with your preferred method. You could use `ukb_df_full_join` which is a thin wrapper around `dplyr::full_join` applied recursively with `purrr::reduce`.

```
ukbxxxx_data <- ukb_df("ukbxxxx")
ukbyyyy_data <- ukb_df("ukbyyyy")
ukbzzzz_data <- ukb_df("ukbzzzz")

ukb_df_full_join(ukbxxxx_data, ukbyyyy_data, ukbzzzz_data)
```

> **Repeated variables.**
>
> The join key is set to “eid” only (default value of the `by` parameter). Any additional variables common to any two tables will have “.x” and “.y” appended to their names. If you are satisfied the additional variables are identical to the original, the copies can be safely deleted. For example, if `setequal(my_ukb_data$var, my_ukb_data$var.x)` is `TRUE`, then my\_ukb\_data$var.x can be dropped. A `dlyr::full_join` is like the set operation union in that all observation from all tables are included, i.e., all samples are included even if they are not included in all datasets.
>
> Repeated variable names **within** UKB datasets are unlikely to occur. `ukb_df` creates variable names by combining a snake\_case descriptor with the variable’s *field code*, *index* and *array*. This should be sufficient to uniquely identify the variable. However, if an *index\_array* combination is incorrectly repeated in the original UKB data, this will result in a duplicated variable name. We observed two instances. The variables were encoded *–0.0*, *–1.0*, *––1.0*, and `ukb_df` created a variable named *var\_0\_0*, *var\_1\_0*, *var\_1\_0*. This is probably a typo that should have been *–0.0*, *–1.0*, *–2.0*, consistent with UKB official documentation describing the field as having 3 values for index. We have provided `ukb_df_duplicated_names` to identify duplicated names within a dataset. This will allow the user to make changes as appropriate. We expect the occurrence of such duplicates will be rare.

## An example fileset

**ukbxxxx.tab**, **ukbxxxx.r**, **ukbxxxx.html**

A minimal example fileset is included with the package, in the subdirectory inst/extdata. This fileset will allow the user to test the the read (`ukb_df`, `ukb_df_field`) and summarise (`ukb_context`) functionality.

```
# To load the example data
path_to_example_data <- system.file("extdata", package = "ukbtools")

df <- ukb_df("ukbxxxx", path = path_to_example_data)

# To create a field code to name key
df_field <- ukb_df_field("ukbxxxx", path = path_to_example_data)
```

The full path to the raw test data can be retrieved with `system.file("extdata", "ukbXXXX.tab", package = "ukbtools")`.

## Exploring primary demographics of a UKB subset

As an exploratory step you might want to look at the demographics of a particular subset of the UKB sample relative to a reference sample. For example, using the `nonmiss.var` argument of `ukb_context` will produce a plot of the primary demographics (sex, age, ethnicity, and Townsend deprivation score) and employment status and assessment centre, for the subsample with data on your variable of interest compared to those without data (i.e. `NA`).

```
ukb_context(my_ukb_data, nonmiss.var = "my_variable_of_interest")
```

It is also possible to supply a logical vector with `subset.var` to define the subset and reference sample. This is particularly useful for understanding a subgroup within the UKB study, e.g., overweight individuals.

```
subgroup_of_interest <- (my_ukb_data$body_mass_index_bmi_0_0 >= 25) 
ukb_context(my_ukb_data, subset.var = subgroup_of_interest)
```

**Primary demographic data for a UKB subset of interest.** The subset is individuals with BMI >= 25; the reference is BMI < 25. Barplots are displayed as proportions, e.g., about 1/3 of all participants who identified as “Chinese” were overweight compared to about 2/3 of all participants who identified as “British”. ukb\_context also allows the user to draw barplots as “stacked” or “side-by-side” bars representing counts, which would reveal there were many more “British” participants (442,698) than there were “Chinese” (1,574).

## Retrieving ICD diagnoses

The International Classification of Diseases (ICD) query functions begin `ukb_icd_`. Type `ukb_icd_` tab to see the family of functions. The full ICD code definition tables are available as datasets (`icd9codes`, `icd10codes`). ICD chapter-level tables describing disease blocks are also available for query as datasets (`icd9chapters`, `icd10chapters`)

*Sample query*

`ukb_icd_diagnosis` takes one or more individual ids and returns a dataframe with a potential message noting ids with no diagnoses

```
ukb_icd_diagnosis(my_ukb_data, id = "0000000", icd.version = 10)
```

*Diagnosis query*

To retrieve the “meaning” of an ICD code use `ukb_icd_code_meaning` accepts one or more ICD codes and returns a dataframe of codes and their associated meanings

```
ukb_icd_code_meaning(icd.code = "I74", icd.version = 10)
```

Search for a class of diseases with a keyword. Supplying multiple keywords as a character vector will return all ICD entries containing *any* of the keywords.

```
ukb_icd_keyword("cardio", icd.version = 10)
```

You can calculate the prevalence of a diagnosis in the UKB study (or a subset of the full sample) using `ukb_icd_prevalence`. The `icd.diagnosis` argument takes a regular expression, and so can also be used to retrieve prevalence of a disease “class”, i.e., the proportion of individuals with *any* diagnosis in the disease class.

```
# ICD-10 code I74, Arterial embolism and thrombosis
ukb_icd_prevalence(my_ukb_data, icd.version = 10, icd.diagnosis = "I74")

# ICD-10 chapter 9, disease block I00–I99, Diseases of the circulatory system
ukb_icd_prevalence(my_ukb_data, icd.version = 10, icd.diagnosis = "I")

# ICD-10 chapter 2, C00-D49, Neoplasms 
ukb_icd_prevalence(my_ukb_data, icd.version = 10, icd.diagnosis = "C|D[0-4].")
```

To retrieve frequency for one or more ICD diagnoses by the levels of a reference variable, e.g., sex (male or female) use `ukb_icd_freq_by`. If the variable is continuous, it is divided into N approximately equal-sized groups (default = 10) within which ICD diagnosis frequency is calculated. `ukb_icd_freq_by` also includes an option `freq.plot` to produce a figure of ICD diagnosis frequency by reference variable. Diagnoses of interest are passed to `icd.code`. The default ICD codes are the WHO top 3 causes of death worldwide (2015): coronary artery disease (CAD), cerebrovascular disease/ stroke, lower respiratory tract infection (LTRI).

```
ukb_icd_freq_by(my_ukb_data, reference.var = "body_mass_index_bmi_0_0", freq.plot = TRUE)
ukb_icd_freq_by(my_ukb_data, reference.var = "sex_0_0", freq.plot = TRUE)
```

**ICD diagnosis frequency by reference variable.** Panel **A** shows frequency of the query diagnoses with respect to BMI; panel **B** with respect to sex. Any diagnoses can be queried with the `icd.code` parameter (default is CAD, Stroke, LRTI)

Setting `freq.plot = FALSE` (default) returns a dataframe of the frequencies. Values for the reference variable group ranges are in the column “group”.

---

## **Genetic metadata**

**UKB genetic data resources**

- UKB Resource 664: Accessing Genetic Data within UK Biobank
- UKB Resource 531: Description of genetic data types

> **Interim release metadata**
>
> The genetic metadata functions were written to retrieve genetic metadata from the phenotype file for the interim genotype release. The associated QC was described in genotyping and quality control and imputation and association. **The fields retrieved became obsolete when the full genotyping results were released at the end of 2017.**
>
> **Full release metadata**
>
> With the release of the full sample (500K individuals) genotypes, sample QC (**ukb\_sqc\_v2.txt**) and relatedness (**ukbA\_rel\_sP.txt**) data are now supplied as separate files. The contents of these files, along with all other genetic files are described in UKB Resource 531.
>
> **Defunct functions**
>
> - ukb\_gen\_meta
> - ukb\_gen\_pcs
> - ukb\_gen\_excl
> - ukb\_gen\_rel
> - ukb\_gen\_het
> - ukb\_gen\_excl\_to\_na
> - ukb\_gen\_write\_plink\_excl
>
> The above functions remain in the ukbtools package but return the message:
>
> ```
> Error: ukb_gen_<name> is defunct.
> See help("ukb_defunct")
> ```
>
> The documentation for `ukb_defunct` includes the information about the change in the way UKB serve the genetic (meta)data.

### New genetic metadata functionality (v0.11.0)

The sample QC file **ukb\_sqc\_v2.txt** has no header. To include a header use `ukb_gen_sqc_names`. From UKB Resource 531: “There are currently 2 versions of this file (UKB ukb\_sqc\_v2.txt) in circulation. The newer version is described below and contains column headers on the first row. The older (deprecated) version lacks the column headers and has two additional Affymetrix internal values prefixing the columns listed below”.

`ukb_gen_sqc_names` handles this if used to include column names in a dataframe of sample QC data. If a character vector of names is needed (e.g. to supply on reading in sample QC data) use the argument `col_names_only = TRUE` and trim if required.

```
# With ukb_sqc_v2.txt read into the dataframe my_sqc_data
my_sqc_data <- ukb_gen_sqc_names(my_sqc_data)

# For a character vector of column names
ukb_gen_sqc_names(col_names_only = TRUE)
```

*Relatedness count*

`ukb_gen_rel_count` now works supplied relatedness file **ukbA\_rel\_sP.txt** with header: ID1, ID2, HetHet, IBS0, Kinship. Supply a dataframe of the relatedness data for a dataframe of counts of degree of relatedness (Duplicates/MZ twins, 1st-degree, 2nd-degree, 3rd-degree).

```
# With ukbA_rel_sP.txt read into the dataframe my_relatedness_data
ukb_gen_rel_count(my_relatedness_data)
```

You can also create a plot of degree of relatedness, e.g., for samples with data on a variable of interest.

```
ukb_gen_rel_count(ukb_relatedness, plot = TRUE)
```

**Degree of relatedness.** KING kinship coefficient ranges > 0.354, (0.177, 0.354], (0.0884, 0.177] and (0.0442, 0.0884] corresponds to duplicate/MZ twin, 1st-degree, 2nd-degree, and 3rd-degree relationships respectively.

*Maximal subset of unrelateds*

There are many ways to remove related individuals from phenotypic data for genetic analyses. You could simply exclude all individuals indicated as having “excess relatedness” and include those “used in pca calculation” (these variables are included in the sample QC data, **ukb\_sqc\_v2.txt**). This list is based on the complete dataset and possibly removes more samples than you need to for your phenotype of interest. Ideally, you want a maximum independent set, i.e., to remove the minimum number of individuals with data on the phenotype of interest, so that no pair exceeds some cutoff for relatedness. `ukb_gen_samples_to_remove` returns a list of samples to remove in order to achieve a maximal set of unrelateds for a given phenotype (default `cutoff > 0.0884` - KING coefficient corresponding to 3rd degree relatedness). For the subset of related pairs (**ukbA\_rel\_sP.txt**) in which both have data on the phenotype of interest use `ukb_gen_related_with_data`.

```
# With ukbA_rel_sP.txt read into the dataframe my_relatedness_data
# and an integer vector samples_with_phenotype of samples who have
# data on the phenotype of interest

ukb_gen_related_with_data(my_relatedness_data, ukb_with_data = samples_with_phenotype)
ukb_gen_samples_to_remove(my_relatedness_data, ukb_with_data = samples_with_phenotype)
```

### Write phenotype and covariate files for genetic analysis

ukbtools includes convenience functions to write phenotype and covariate files for BGENIE and PLINK.

**BGENIE** phenotype and covariate files are space-delimited, include column names, and have missing values coded as -999. They must also be in .sample file order. The sample file is downloaded with your bulk genotype data and described in UKB Resource 531. The sample file is a rectangular file with two header lines. Using `ukb_gen_read_sample` will read it in correctly and label the columns. `ukb_gen_write_bgenie` sorts input data to match .sample file id order and writes the data to disk.

```
# Read .sample file supplied with bulk genetic data
my_sample_file <- ukb_gen_read_sample("path/to/sample_file")

# Write a BGENIE format phenotype or covariate file
ukb_gen_write_bgenie(
    my_ukb_data, 
    path = "path/to/bgenie_input_file", 
    ukb.sample = my_sample_file, 
    ukb.variables = c("variable1", "variable2", "variable3")
)
```

The BGENIE usage page uses the example files example.pheno and example.cov - it is not clear whether the suffixes are obligatory. Use them to be safe.

**PLINK** phenotype and covariate files are either space- or tab-delimited, column names are optional, first two columns must contain family ID and individual ID respectively, and missing values are “normally expected to be encoded as -9” but also “nonnumeric values such as ‘NA’ … (are) treated as missing”. `ukb_gen_write_plink` writes a space-delimited file with column names, UKB ID is automatically written to column 1 and 2 and labelled FID IID, and missing values are coded as `NA`. The missing value to be used can be changed with the `na.strings` argument. See PLINK standard data input for further details.

```
# Write a PLINK format phenotype or covariate file

ukb_gen_write_plink(
    my_ukb_data, 
    path = "path/to/plink_input_file", 
    ukb.variables = c("variable1", "variable2", "variable3")
)
```

**PLINK** does not require that individuals in phenotype and covariate files are in any particular order, but you may want to reconcile the individuals you include in your analysis with those in the fam file (from your hard-called data). Read the fam file into R with `ukb_gen_read_fam`

*Sample exclusions*

**BGENIE** does not have an option to read/remove exclusions. You can replace data values in the phenotype with `NA` where the individual is to-be-excluded based on genetic metadata considerations. Writing the updated variable to your phenotype file (with the supplied write functions), effectively excludes the individuals from any analysis.

**PLINK** `--remove` takes a space- or tab-delimited file with family IDs in the first column and individual IDs in the second column, without column names. See PLINK input filtering for further details. (The missing value approach described above also works for PLINK.)

Use the sample QC file (**ukb\_sqc\_v2.txt**) to determine to-be-excluded samples.

## Acknowledgements

This research has been conducted using the UK Biobank Resource, Application 13427.
