# Supplementary figures and images for: ukbtools: An R package to manage and query UK Biobank data
Source: PLoS One. 2019 May 31;14(5):e0214311. doi: 10.1371/journal.pone.0214311 (PMC6544205; doi:10.1371/journal.pone.0214311)

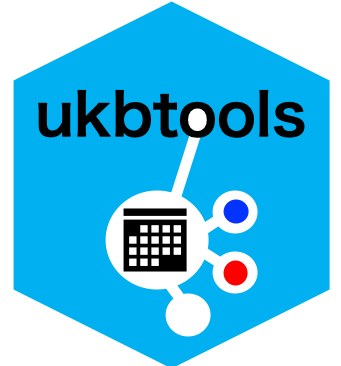

Supplement: S3 File — This bundle contains ukbtools v0.11.3. (TAR.GZ) [file pone.0214311.s003.tar.gz › ukbtools/man/figures/logo.png]

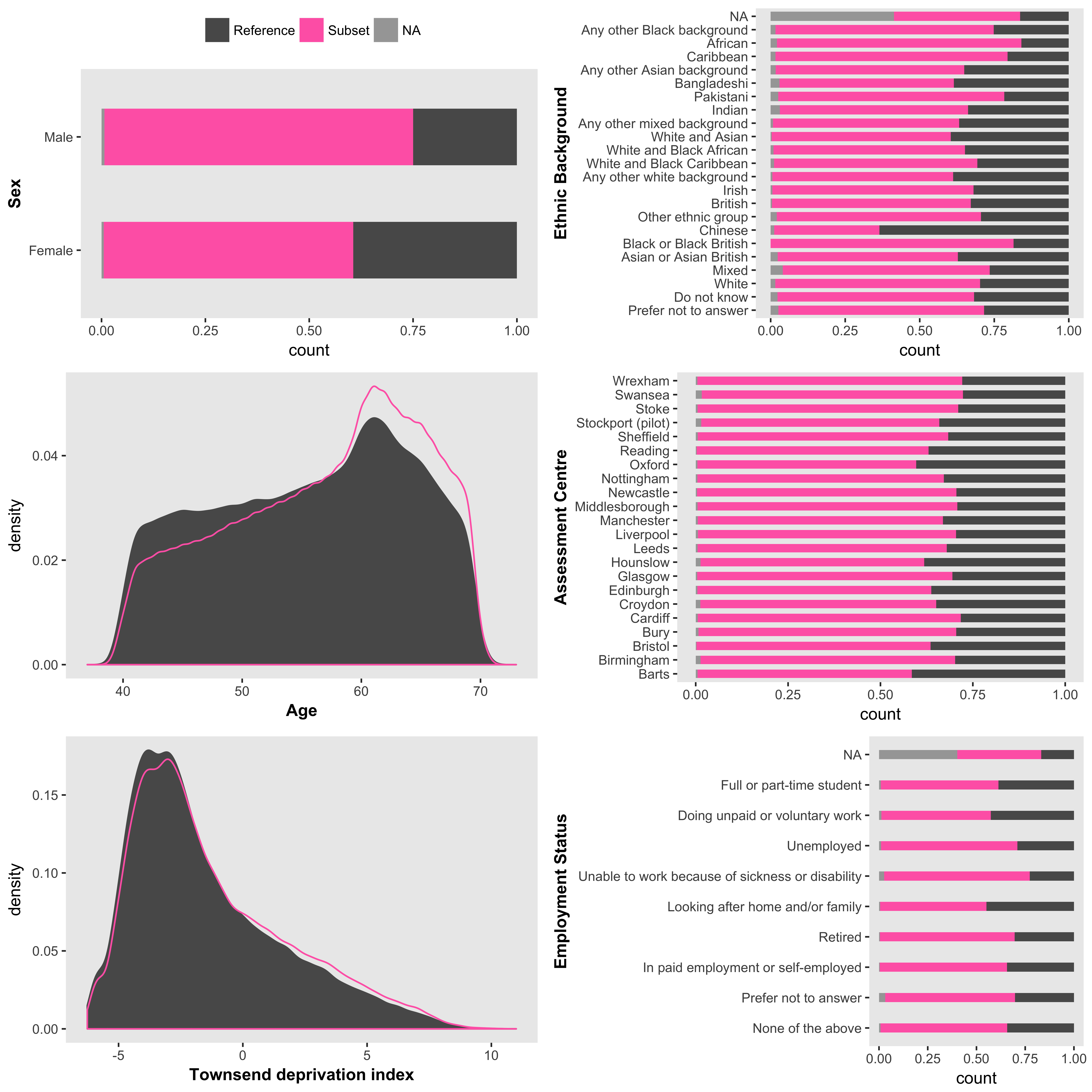

Supplement: S3 File — This bundle contains ukbtools v0.11.3. (TAR.GZ) [file pone.0214311.s003.tar.gz › ukbtools/vignettes/img/ukb_context_fill_111017.png]

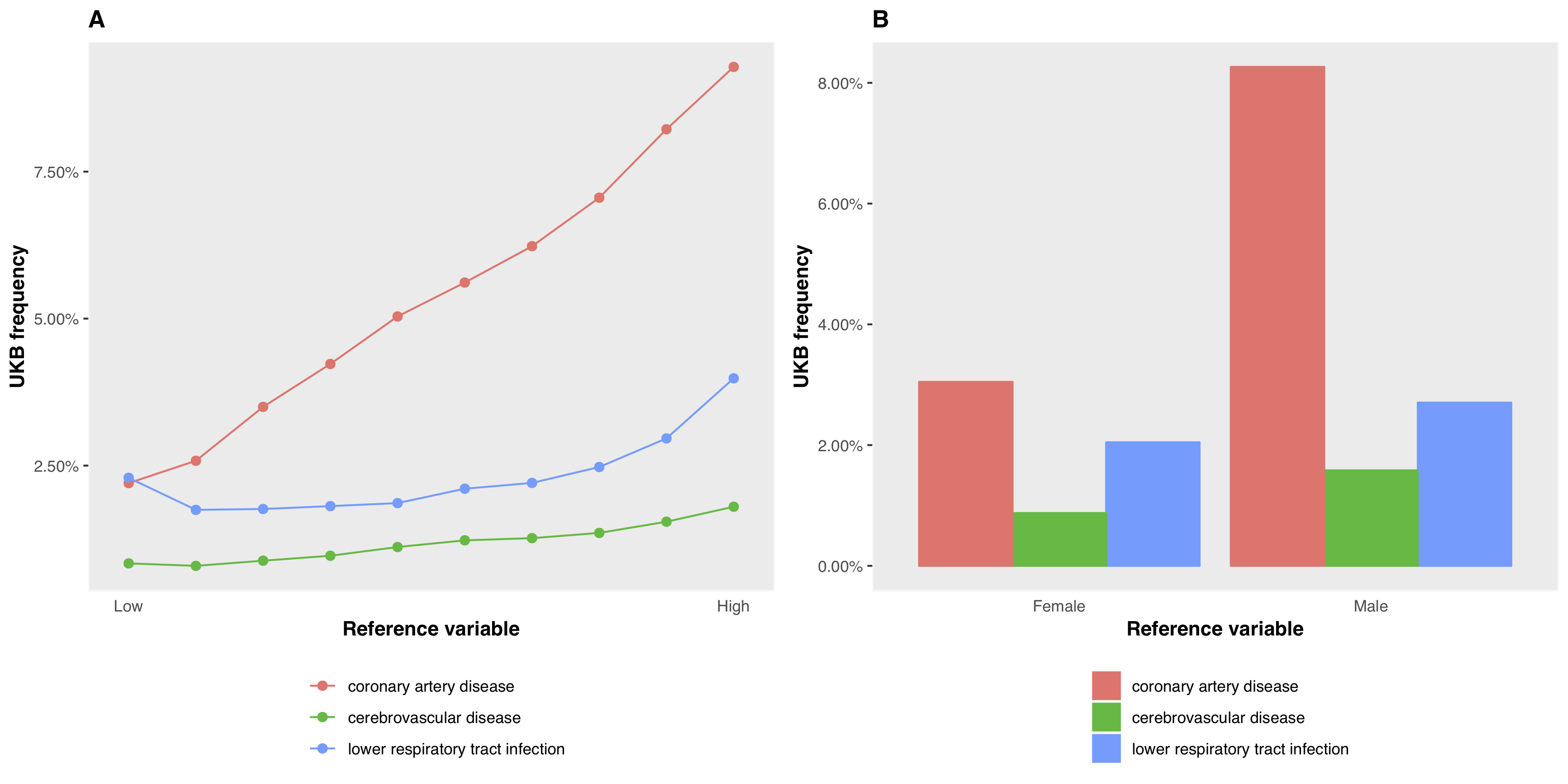

Supplement: S3 File — This bundle contains ukbtools v0.11.3. (TAR.GZ) [file pone.0214311.s003.tar.gz › ukbtools/vignettes/img/ukb_icd_freqby.png]

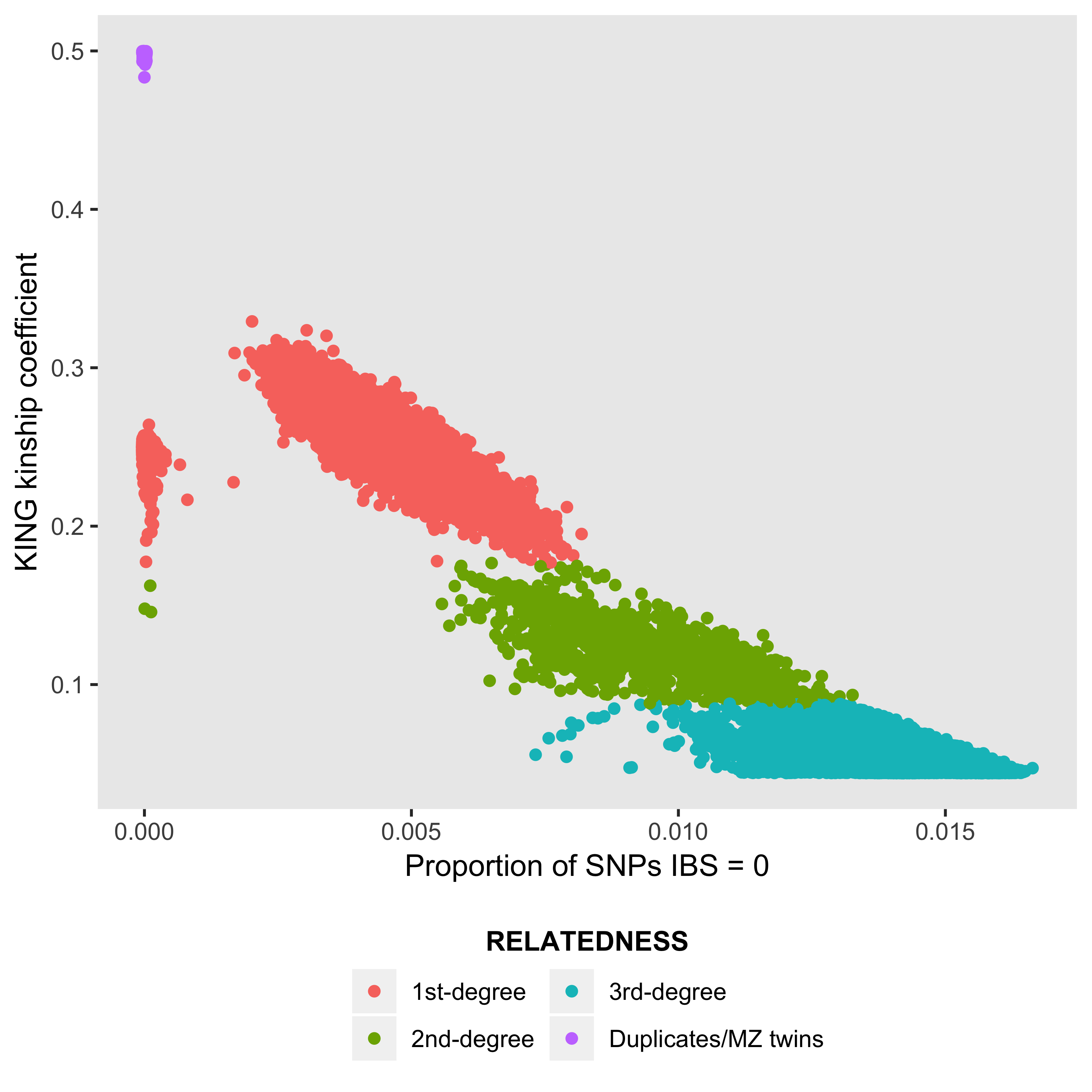

Supplement: S3 File — This bundle contains ukbtools v0.11.3. (TAR.GZ) [file pone.0214311.s003.tar.gz › ukbtools/vignettes/img/ukb_relatedness.png]
